# New criteria for sustainable land use planning of metropolitan green infrastructures in the tropical Andes

**DOI:** 10.1007/s10980-024-01911-2

**Published:** 2024-05-28

**Authors:** María José LaRota-Aguilera, Emmanuel Zapata-Caldas, Oscar Buitrago-Bermúdez, Joan Marull

**Affiliations:** 1 Institute of Regional and Metropolitan Studies of Barcelona, Barcelona, Spain; 2 Centro Internacional de Agricultura Tropical (CIAT), University of Valle (Primary), Valle del Cauca, Colombia; 3 University of Valle, Valle del Cauca, Colombia; 4 Minuartia, Barcelona, Spain

**Keywords:** Integrated socio-ecological assessment, Social metabolism, Ecosystem services, Biocultural landscapes, Landscape sustainability, Agroforestry mosaics

## Abstract

**Context:**

Urbanization is rapidly increasing worldwide, with about 60% of the global population currently residing in cities and expected to reach 68% by 2050. In Latin America's tropical Andes region, managing these changes poses challenges, including biodiversity loss and vulnerability to climate change.

**Objectives:**

This study assesses urban growth and agricultural intensification impacts on the ecological functionality of metropolitan green infrastructures and their capacity to provide ecosystem services using a landscape sustainability and sociometabolic approach. Specifically, it aims to identify landscape configurations promoting socio-ecological sustainability amidst rapid urbanization.

**Methods:**

A landscape-metabolic model (IDC) was applied to evaluate the interactions between land use changes and ecosystem functions in the metropolitan region of Cali.

**Results:**

Agricultural intensification and industrialization, coupled with uncontrolled urban growth, have significantly transformed the landscape, posing threats to its sustainability. The prevailing biocultural landscapes hold a substantial potential to provide essential ecosystem services to the metropolis. The IDC offers an approach that utilizes a land cover map and agricultural production/metabolism data to calculate an indicator closely related to ecosystem services and multifunctionality.

**Conclusions:**

The IDC model stands out for efficiently capturing landscape dynamics, providing insights into landscape configuration and social metabolism without extensive resource requirements. This research highlights the importance of adopting a landscape-metabolic and green infrastructure framework to guide territorial policies in the tropical Andes and similar regions. It stresses the need for informed land use planning to address challenges and leverage opportunities presented by biocultural landscapes for regional sustainability amidst rapid urbanization and agricultural expansion.

## Introduction

Urban centres and their interactions with peri-urban and rural areas have historically been pivotal in society's development, with farmland and natural ecosystems supplying essential resources (Steel 2008). However, the Industrial Revolution introduced two major socio-ecological changes: urbanization and agricultural intensification (Krausmann et al. 2008; Swyngedouw and Heynen 2003). Coupled with current economic and technological models, population growth has led to increased resource consumption in cities, impacting water, energy, materials, and food (Balatsky et al. 2015; Krausmann et al. 2009). This transformation from natural and low-intensity agriculture to urban and high-intensity agriculture has resulted in biodiversity and ecosystem service losses (Elmqvist et al. 2013), disrupted water flows (Hibbs and Sharp Jr 2012), and escalated global greenhouse gas emissions (IPCC 2014; Vermeulen et al. 2012).

At local levels, urbanization and agricultural intensification affect ecosystems' ability to provide essential services and support life (Brondizio et al. 2019). Moreover, under current climate change scenarios, biodiversity and ecosystem service loss heighten vulnerability to climate impacts (Burak Güneralp et al. 2013; IPCC 2014; McDonald et al. 2013). Despite this reality, the global population is projected to reach 9.8 billion by 2050, with 68% residing in urban areas (United Nations 2019). The impacts of this trend will disproportionately affect low- and lower-middle-income regions, where rapid urbanization is expected, potentially straining resilience and adaptation capacity (IPCC 2014).

This complex scenario presents unprecedented challenges for urban–rural relationships and land-use planning in metropolitan regions, requiring comprehensive and integrated approaches to find solutions (Yacamán-Ochoa et al. 2020). In this sense, the concept of green infrastructure has gained importance in understanding the role of metropolitan open spaces in sustaining urban environments (Benedict and McMahon 2002). However, gaps persist in how to effectively implement green infrastructure for biodiversity conservation and ecosystem service provision (Chatzimentor et al. 2020; Demuzere et al. 2014; Vásquez et al. 2019).

Notably, most advances in green infrastructure have occurred in the Global North (Chatzimentor et al. 2020; European Commission 2013; Slätmo et al. 2019), while the Global South, including Latin America's tropical Andes, lags (Pauleit et al. 2021). In these regions, land-use changes driven by agricultural intensification and resource exploitation pose unique challenges. The resulting rural-to-urban migration is exacerbated by political instability, corruption, and armed conflicts, leading to disorganized metropolitanization and inadequate urban planning (Aide et al. 2019; Canales and Canales Cerón, 2013; Angotti 1996).

Therefore, tropical Andean countries face a critical choice: sustain their natural ecosystems or pursue current economic development models. The metropolitan region of Cali, Colombia, exemplifies this dilemma, experiencing significant socioeconomic and land-use changes driven by agro-industrial practices and rural migration (Delgadillo-Vargas 2014; Marull et al. 2017; Martínez-Toro and Patiño-Gómez 2015; Centro Nacional de Memoria Histórica 2014; Uribe-Castro 2017).

To improve landscape sustainability and address contemporary challenges, it's crucial to understand how landscape patterns influence ecosystem services and human wellbeing (Wu 2006, 2013). We propose that this endeavor demands for integrated and transdisciplinary approaches that explore the relationship between different landscape-metabolic configurations within metropolitan regions and their capacity to provide ecosystem services.

This article employs a landscape-metabolism model (Marull et al. 2019, 2018) to comprehensively evaluate the ecological functionality of Cali's metropolitan green infrastructure. The study pursues three main objectives: assessing the impacts of agricultural intensification and urbanization on the green infrastructure's ecological functions and services, analysing the relationship between these configurations and their ability to supply ecosystem services, and guiding future land use policies for a functional metropolitan green infrastructure in the region. By adopting a landscape sustainability perspective, this study proposes new criteria for sustainable land use planning in metropolitan green infrastructures, ensuring that these regions can meet present and future needs without compromising the integrity of their ecosystems.

In the following sections, we provide a brief context for the case study, outline the methodologies employed, present the landscape-metabolic assessment results, and discuss the emerging opportunities and challenges posed by agricultural landscapes for the sustainability of Cali's metropolitan region. We then assess the relevance of our findings and the potential of adopting a green infrastructure framework to shape future land use policies in the tropical Andes.

## Methodology

### Case study

**Fig. 1:**
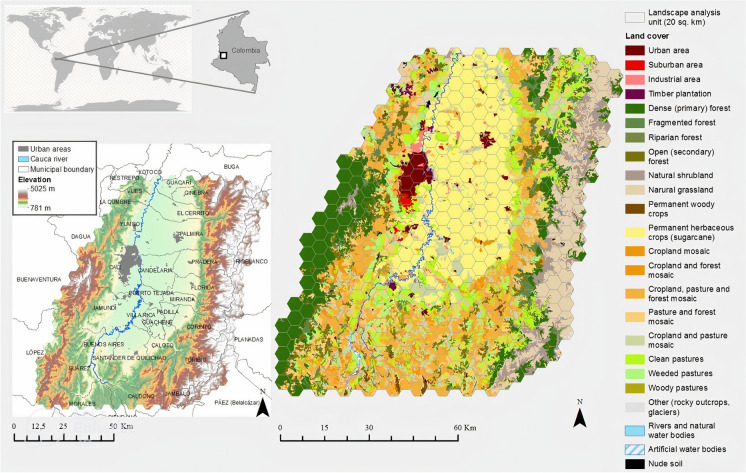
Location, administrative limits, and land cover map of the metropolitan region of Cali. Source: Corine Land Cover Map for Colombia 2012 (level 3 legend)

Currently, the metropolitan region of Cali is not considered an official administrative entity. There have been some initiatives to formally consolidate it, mainly based on capital criteria dictated by the predominant role of agribusiness in the region; however, they have not thrived (Martínez-Toro and Patiño-Gómez 2015; Urrea-Giraldo and Candelo-Álvarez 2017). Therefore, this study considers the Upper Cauca River Valley (henceforth UCRV) as the territorial reference for the metropolitan region and the study area.^1^ The UCRV limits were defined based on three criteria: i) the third metropolitan crown (Martínez-Toro and Patiño-Gómez 2015); ii) the limits of the hydrographic sub-basins in which the urban centres of the third metropolitan crown are located; and iii) the areas of influence of sugarcane cultivation, given their economic importance for the region (Fig. 1). The population in the UCRV is approximately 3′635.573 people, and 2′172,527 people live in the urban area of the city district of Cali (DANE 2018).

The UCRV is located within the geographic valley of the Cauca River (448,000 ha), between the Central and Western Mountain ranges of the Andes, in southwest Colombia (Fig. 1). The study area occupies 1′004,000 hectares and includes elevations between 800 and 5,000 m above sea level. Originally, this biogeographic unit was made up of various ecosystems, including tropical dry forests, tropical pastures, high Andean forests, and paramos. However, currently, 19.2% of the UCRV is covered by sugar cane monocultures (192,742 ha); 31,1% by mixed crops that include coffee, banana, and fruit trees, in mosaics with natural areas and pastures (312,682 ha); 13% by natural and planted open pastures (130,547 ha); 32.5% by forests and other natural areas -among which are the last enclave of tropical dry forest and paramos- (198,659 ha); and 2.4% by urban and suburban spaces built (23,954 ha) (Fig. 1).

In the UCRV, two key land cover changes have occurred: i) the shift from clean pastures, cropland (including sugarcane), and seminatural transition areas to low-density urban zones, and ii) the expansion of sugarcane crops across the flat region, previously a mosaic of pastures and mixed crops, dating back to the mid-nineteenth century, profoundly altering the landscape (Delgadillo-Vargas 2014). Cali's rapid growth is influenced by three factors: its designation as the Valle del Cauca department capital in 1910, concentrating public services; the growth of its industrial sector (Vásquez 2018); and over five decades of violence and internal conflict, making the city a major destination for internal refugees (140,751 individuals between 1985 and 2014; Centro Nacional de Memoria Histórica 2015). This case study is essential for understanding these complex dynamics.

### Landscape ecological metrics

The landscape's functional structure evaluation was based on the adaptation of the Corine Land Cover map of land cover at a scale of 1:100,000 for Colombia, considering level 3 of the legend (Fig. 1). The reference year is 2015 since it is the most recent year with relevant information. This map was updated with supervised classification and interpretation of aerial photographs for 2013 and 2016 by the authors to correct areas with high cloudiness. The final coverage legend includes 22 classes and is shown in Table 1.

**Table 1:** Land cover and surface distribution in the metropolitan region of Cali

Typology	Land cover	Reclassification	Area (ha)	%
Forest and seminatural areas	Rocky outcrops	Others (rocky outcrops, glaciers)	257.81	0.0%
	Natural sandy areas	Others (rocky outcrops, glaciers)	287.51	0.0%
	Glacial and snow zones	Others (rocky outcrops, glaciers)	66.12	0.0%
		*Total others*	*611.44*	*0.1%*
	Natural shrublands	Natural shrublands	39627.44	3.9%
	Natural grasslands (Paramo)	Natural grasslands (Paramo)	87665.07	8.7%
	Dense forest	Dense forest	117687.3	11.7%
	Riparian forest	Riparian forest	2487.41	0.2%
		*Total primary forest*	*120174.7*	*12.0%*
	Fragmented forests	Secondary forest	29330.82	2.9%
	Secondary vegetation or in transition	Secondary vegetation or in transition	48542.91	4.8%
		*Total secondary forest*	*77873.73*	*7.8%*
		*Total forest and seminatural areas*		*32.5%*
Agricultural land	Permanent woody crops	Permanent woody crops	3635.33	0.4%
	Permanent herbaceous crops	Sugarcane plantations^[1]^	192742.2	19.2%
		*Total permanent crops*	*196377.5*	*19.6%*
	Pasture and forest mosaic	Pasture and forest mosaic	75165.72	7.5%
	Cropland mosaic	Cropland mosaic	1922.57	0.2%
	Cropland and forest mosaic	Cropland and forest mosaic	18478.08	1.8%
	Cropland, forest, and pasture mosaic	Cropland, forest, and pasture mosaic	152551.3	15.2%
	Cropland and pasture mosaic	Cropland and pasture mosaic	64564.77	6.4%
		*Total agricultural mosaics*	*312682.5*	*31.1%*
	Wooded pastures	Wooded pastures	4673.74	0.5%
	Weeded pastures	Weeded pastures	41797.32	4.2%
	Clean pastures	Clean pastures	84076.29	8.4%
		*Total pastures*	*130547.3*	*13.0%*
	Timber plantations	Timber plantations	3238.40	0.3%
		*Total agricultural land*		*64.0%*
Bodies of water	Rivers (50 m)	Rivers and natural water bodies	2569.59	0.3%
	Artificial water bodies	Artificial water bodies	1637.38	0.2%
	Lagoons, lakes, swamps, and natural swamps	Rivers and natural water bodies	527.56	0.1%
		*Total water*	*4734.54*	*0.5%*
Build-up areas	Continuous urban fabric (Urban areas)	Urban areas	16228.67	1.6%
	Discontinuous urban fabric (Suburban areas)	Urban areas	3447.70	0.3%
	Industrial or commercial areas (Industrial area)	Urban areas	4278.26	0.4%
		*Total urban and industrial areas*	*23,954.63*	*2.4%*
Other	Nude and degraded soils	Nude soils	854.29	0.1%
Total			1,004,000	100.0%

Source: Own elaboration based on the Corine Land Cover Map for Colombia

The study area was divided into 502 analysis units (hexagons), each of 20 km^2^ (2,000 ha) (Fig. 1), for which four indicators were calculated: i) the Shannon Index ii) the Ecological Connectivity Index iii) the Human Appropriation of Net Primary Productivity; and iv) the Intermediate Disturbance Complexity (IDC) model. Each of these landscape metrics is described below.

#### *Shannon index (H')* (Shannon and Weaver 1948)

Characterises the landscape structure as a function of the land cover heterogeneity

$$H= \sum_{i=1}^{j=k}pj \bullet Log pi$$

For its calculation, eight land covers (*j*) considered potential habitats for biodiversity (forest, shrubland, grassland, heterogeneous crops, sugarcane, pastures, bodies of water, rocky outcrops, sandy areas) and one "no habitat" category grouping urban and industrial areas, degraded land, and road infrastructure, were defined. Thus, *Pji* is the proportion of land cover *j* in hexagon *i.* The H' values range from 0 to 1, with 0 being a homogeneous landscape with a single predominant cover and 1 being a theoretical landscape with many land cover classes distributed equitably.

#### *Ecological connectivity index (ECI)* (Marull and Mallarach 2005)

Assesses the functionality of the landscape in terms of the ecological connectivity between related land covers (Pino and Marull 2012). ECI was calculated through a cost-distance model based on an affinity matrix considering 7 types of 'functional ecological areas' (i.e., forest, shrubland, grassland or paramo vegetation, agroforestry mosaics, crops, pastures, and sugar cane), and an impact matrix considering the 'anthropogenic barriers' (i.e., urban areas, infrastructures). The selection criteria for functional ecological areas, the coefficients, and the type of anthropogenic barriers were obtained from a review of the literature and local experts' knowledge. Ecological connectivity is calculated for each of the different functional ecological areas:

$${\text{ECI}}_{\text{b}}=10-9\text{ In }\left(1+{\text{X}}_{\text{i}}\right)/\text{In }{\left(1+ {\text{X}}_{\text{t}}\right)}^{3}$$

where X_*i*_ is the value of the sum of the cost distance per pixel and X_t_ is the maximum theoretical cost distance.

The total ecological connectivity values are calculated from the values obtained for each type of functional ecological area:

$$\text{ECI }=\sum ECIb /m$$

where *m* is the absolute number of functional ecological areas considered. The highest values, in a range of 0 to 10, represent high ecological connectivity.

#### Human appropriation of net primary production (HANPP)

Measures the disturbance exerted by society on a particular ecosystem (Haberl et al. 2007) as a function of the degree to which humans modify the amount of NPP available to other species, fundamentally through two processes: the removal of a portion of the NPP as food., fibre and material of use for society (NPPh) and the change in land cover (∆NPPLu) (Haberl et al. 2007; Krausmann et al. 2013). Recent studies suggest that the indicators associated with HANPP provide key information for planning and evaluating ecosystem services (Mayer et al. 2021). HANPP considers NPP as the net amount of biomass produced by autotrophic organisms, in this case, plants, which constitute the primary energy source for the rest of the food chain for one year. In this sense, HANPP measures the:

$$\begin{array}{c}{\text{HANPP}}_{\text{i}}= {\Delta \text{ NPP}}_{\text{In}}+ {\text{NPP}}_{\text{h}}\\ {\Delta \text{NPP}}_{\text{Lu}}= {\text{NPP}}_{0}- {\text{NPP}}_{\text{act}}\end{array}$$

In turn, ∆NPP_Lu_ is the difference between the potential NPP (Krausmann et al. 2013; available at https://www.aau.at/blog/global-hanpp-2000/) and the actual NPP (NPP_act_) based on disaggregated agricultural production data for the region obtained from the 3rd National Agricultural Census (2014) and based on (Guzmán-Casado et al. 2014).

To obtain the HANPP value per unit of analysis (hexagon), HANPP values (P) for each land cover *i* were multiplied by a w_*i*_ coefficient representing the proportion of land cover *i* in each hexagon. HANPP units are presented in Tons of C / ha.

$${\text{HANPP}}_{\text{hex}}= {\sum }_{\text{i}=1}^{\text{k}}\text{wiPi}$$

#### Intermediate disturbance complexity (IDC)

The IDC model proposed by Marull et al. (2016a, b) transfers the concept of intermediate disturbance in natural ecosystems (Cornell 1978) to human-transformed landscapes (e.g., agroecosystems). The IDC argues that heterogeneous and well-connected land covers, with intermediate levels of agricultural activity, reflect an interaction between landscape complexity and energy availability that constitutes an agroecological matrix capable of harbouring great biodiversity (Loreau 2000; Tscharntke et al. 2012). Therefore, the IDC assesses the landscapes' capacity to host biodiversity and provide ecosystem services (Marull et al. 2019, 2018, 2016b).

The IDC model is calculated from the biomass available for other species (1-HANPP / 100) and the complexity of the landscape (Le). Le describes in a combined way the patterns (L) and processes (ECI) of the landscape (Marull et al. 2018).

$$\text{Le}= \left(\text{aL}+\text{b}\frac{\text{ECI}}{10}\right) 1 /\left(\text{a}+\text{b}\right)$$

where *a* and *b* are the canonical coefficients for the ortorthogonalization of the indices.

$$\text{IDC}=\text{Le }\left(1-\text{HANPP }/ 100\right)$$

### Ecosystem services

The UCRV landscapes' supply and demand of ecosystem services were assessed by Tabares-Mosquera et al. (2020) and based on the Common International Classification of Ecosystem Services (CICES) v.4.3 typology (Haines-young and Potschin 2012), which is associated with the defined ecosystem service categories: provisioning, regulation and cultural, of the Millennium Ecosystem Assessment (2005). Twenty-one ecosystem services were selected that appropriately fit the spatial scale of the Cali metropolitan phenomenon (Table 2).

**Table 2:** Ecosystem services considered in the analysis

Section	Division	Code	Group
Provisioning	Nutrition	1.1.1	Biomass
		1.1.2	Water
	Materials	1.2.1	Biomass
		1.2.2	Water
		1.2.3	Metallic and non-metallic abiotic materials
	Energy	1.3.1	Biomass
		1.3.2	Renewable abiotic source
		1.3.3	Non-renewable abiotic source
Regulation and support	Regulation of waste, toxic substances, and other nuisances	2.1.1	Mediation by living systems
	Flow regulation	2.2.1	Mass flows
		2.2.2	Liquid flows
		2.2.3	Gas/air flows
	Maintenance of physical, chemical, and biological conditions	2.3.1	Maintenance of the life cycle, habitat, and protection of the gene pool
		2.3.2	Control of pests and diseases
		2.3.3	Soil formation and composition
		2.3.4	Maintenance of the chemical composition of water
		2.3.5	Atmospheric composition and climate regulation
Cultural	Physical and intellectual interaction	3.1.1	Physical experience
		3.1.2	Intellectual and representative
	Symbolic and spiritual interaction	3.2.1	Spiritual or emblematic
		3.2.2	Existence and natural intrinsic value

Source: Tabares-Mosquera et al. 2020, adapted from Haines-young and Potschin 2012

Since the land cover pattern is one of the most critical factors affecting the ability of a landscape to provide ecosystem services (Burkhard et al. 2009), the land cover map was used as the basis for quantifying multifunctionality and the capacity to provide ecosystem services of the landscapes that make up the UCRV. Ecosystem services were assessed using an expert-knowledge approach, given the region's incipient developments of ecosystem service mapping (Jacobs and Burkhard 2017). An interdisciplinary group of Twenty-seven experts was selected to evaluate the capacity to supply and demand ecosystem services for each land cover type (Tabares-Mosquera et al. 2020). The experts had to: i) be specialists in at least one of the following groups of land use: agricultural production areas, forests, seminatural areas, humid areas, or artificial areas; ii) be knowledgeable about the study area; and iii) be affiliated with public administrative institutions (mayors and governments), universities or private research centres.

The experts qualitatively evaluated the capacity of land covers to supply or demand ecosystem services, based on a six-class Likert scale: not relevant (0); very low (1); low (2); medium (3); high (4); very high (5) (Albert et al. 2016; Koschke et al. 2012). Experts' responses were averaged to obtain the supply and demand matrices. Finally, two criteria were established to evaluate the ecosystem function of each land cover. The first criterion, 'capacity', is defined as the long-term ability to provide different ecosystem services (Jacobs and Burkhard 2017). Capacity was calculated from the difference between ecosystem services supply and demand and can take values between -5 (very low capacity) and 5 (very high capacity) (Burkhard et al. 2014, 2009). The second criterion, 'multifunctionality', accounts for the number of ecosystem services of various categories (i.e., provisioning, regulation, and cultural) offered by each land cover class(Tabares-Mosquera et al. 2020). The Multifunctionality can take values from 0 (minimum) to 5 (maximum).

The Ecosystem Services Capacity (ESC*j*) and Multifunctionality of Ecosystem Services (MF*j*) for each hexagon (*j*) were calculated as the sum of each land cover capacity or multifunctionality within *j*, and weighted by the proportion of land cover *I in j* (*Pij)*.

$$\begin{array}{c}{\text{ESC}}_{j}= {\sum }_{j=1}^{k}ESCi \bullet Pij\\ {\text{MF}}_{j}= {\sum }_{j=1}^{k}ESCi \bullet Pij\end{array}$$

### Statistical analyses

To assess the contribution of the different land covers to the expression of the IDC, a step-wise multiple regression model (MRM) was performed where IDC was the dependent variable, and the 22 land cover classes of the area were the predictor variables. Additionally, a linear regression analysis was performed to explore the relationship between IDC and the ESC based on the results obtained for each unit of analysis (hexagons; n = 502).

## Results and discussion

### Ecological functions and services of the metropolitan green infrastructure

#### Landscape metrics

**Fig. 2:**
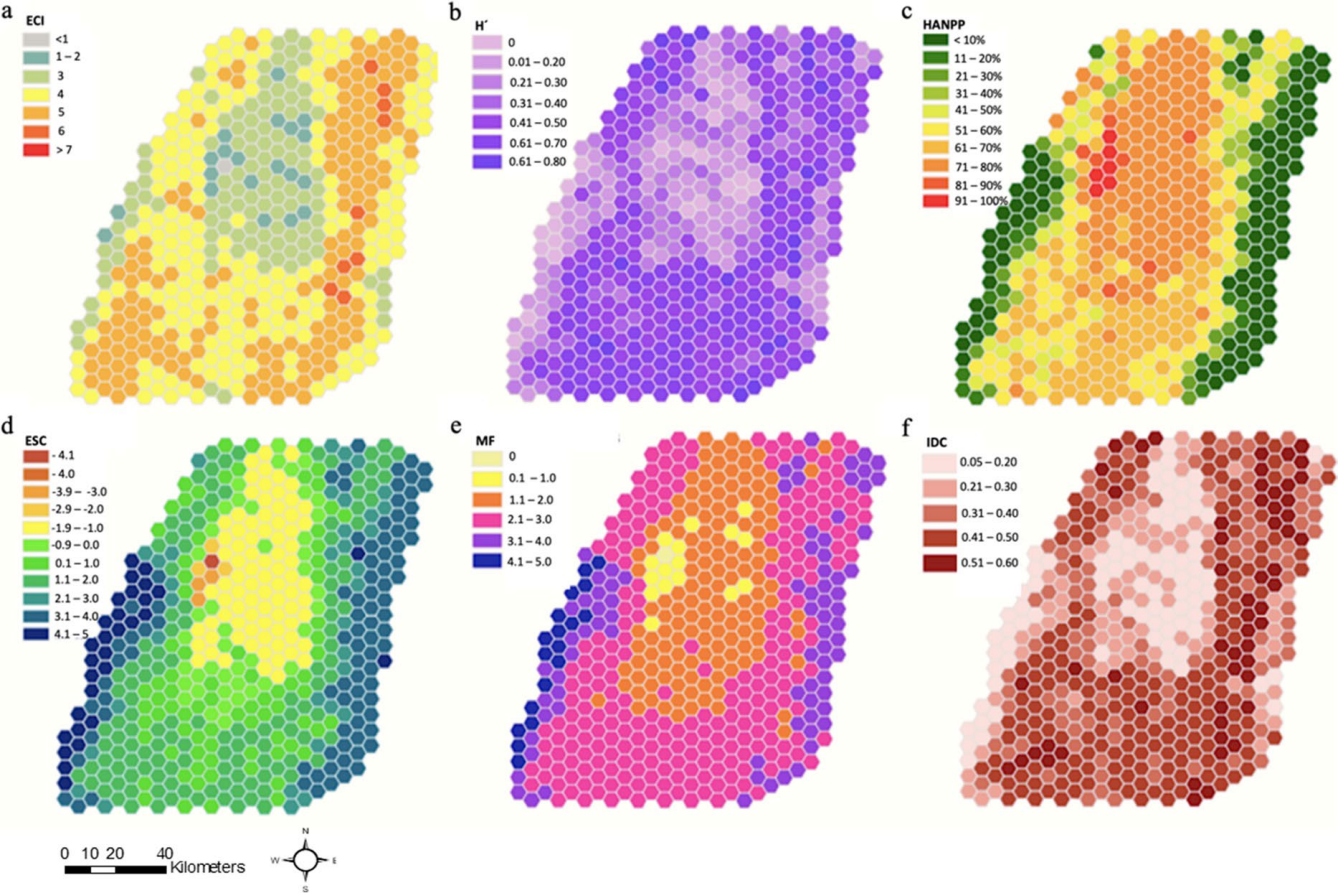
Ecological functions and services of the metropolitan region of Cali. Ecological Connectivity Index -ECI (**a**); Shannon Index -H' (**b**); Human Appropriation of Net Primary Production -HANPP (**c**); Ecosystem Services Capacity -ESC (**d**); Multifunctionality of Ecosystem Services -MF (**e**), and Intermediate Disturbance Complexity -IDC (**f**)

The results indicate the highest levels of anthropogenic disturbance in the flat zone of the UCRV, where the main urban and industrial centres, road infrastructure, and sugarcane monoculture are located (HANPP > 61%; Fig. 2c). HANPP shows the existence of a gradient in the intensity of agricultural land use, which decreases with elevation as we move to the southern zone of the metropolitan region. This spatial pattern of anthropic disturbance would be associated with a disruption of ecological connectivity (ECI) in the flat zone of the study area (Fig. 2a). This area is also characterised by low landscape heterogeneity (H') (Fig. 2b). On the contrary, a strip of higher connectivity (Fig. 2a) and heterogeneity (Fig. 2b) stands out on the slopes of both mountain ranges, which also coincide with intermediate levels of anthropic disturbance (Fig. 2c).

**Fig. 3:**
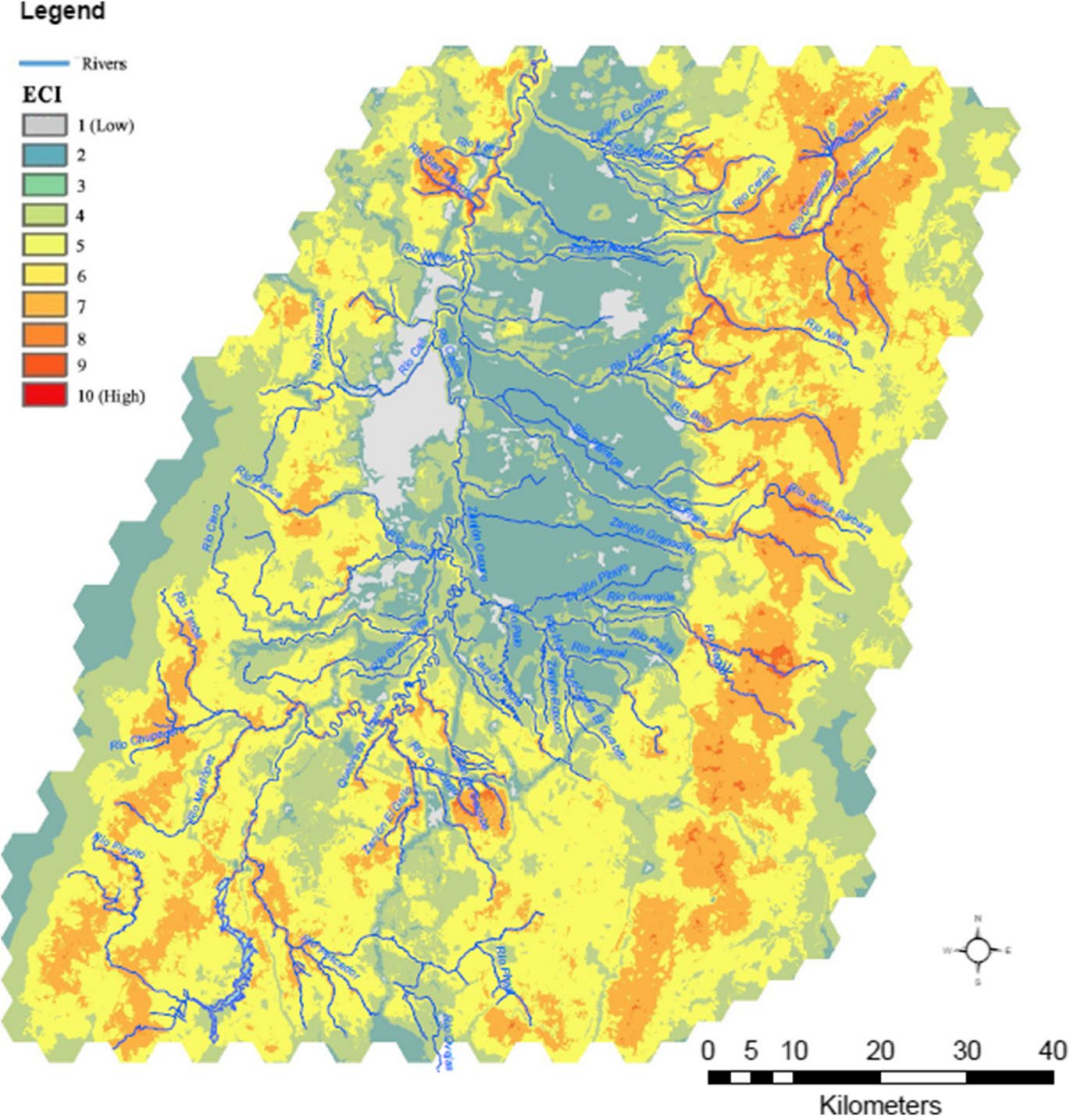
Ecological Connectivity Index (ECI) of the metropolitan region of Cali

The ECI levels reflect an overall ecological disconnection between the valley and key natural areas (i.e., forests, natural shrubs, natural grasslands; Fig. 1) mainly located at high elevations of both Andean mountains (Fig. 3). Even though the ECI model considers the hydrological network a fundamental element for ecological connectivity, the connective function exerted by rivers, especially the Cauca River, is very subtle. This situation contrasts with the one found in other metropolitan regions of the world, where riparian ecosystems play a key role in maintaining ecological connectivity even within highly antrhopogenized areas (Dupras et al. 2016; Padró et al. 2020a).

The low connectivity associated with the hydrological network (Fig. 3) could be explained by the low ecological affinity between forests and pastureland covers. In this region, pastures play a critical and controversial role in the landscape functioning of this region and the socioecological sustainability. For instance, many clean pastures adjacent to rivers result from deforestation driven by extensive cattle ranching and land speculation (Garcia Corrales et al. 2019; Rodríguez Eraso et al. 2013; Zuluaga et al. 2021). The land cover changes associated with pastures establishment is a phenomenon risking homogenising the UCRV and overall mountain landscapes in the tropical Andes. Similarly, riparian natural land covers have also been pushed to the limits by sugarcane monoculture (Ayala-Osorio 2019; Delgadillo-Vargas 2014; Pérez-Rincón 2018). Additionally, although beyond the scope of this work, illegal human settlements and resulting water pollution are critical issues for the Cauca River (Holguin Gonzalez and Goethals 2010). All these conditions have limited the river's ecological connectivity potential. These findings are aligned with the water use conflict between agricultural demands and urban water consumption (Pérez-Rincón et al. 2011) and offer a comprehensive analysis of the larger river basin, complementing prior research conducted at local and sub-basin scales which identified desertification, high scarcity indexes (Pérez-Rincón et al. 2011), and river reversion (Delgadillo-Vargas 2014; Marull et al. 2017) in the region.

#### Landscape-metabolism model (IDC)

The IDC model serves as a valuable tool for exploring landscape sustainability by investigating the regional biophysical connections between humans and nature (Wu 2021). It does so by elucidating the landscape-metabolic relationships that reflect the complex socio-ecological processes occurring in this rapidly evolving metropolitan region. The relationship between land cover types and the Intermediate Disturbance Complexity (IDC) within the study area showed that the overall model fit statistics, including the multiple R-squared (0.7433) and adjusted R-squared (0.7381), indicating that approximately 74.33% of the variance in IDC values could be explained by the land cover variables included in the analysis. Similarly, the results revealed notable associations between specific land cover types and IDC values (Table 3). Specifically, sugarcane plantations, urban areas, and natural areas (dense forest, natural grasslands) have a significant (p < 0.050) negative relationship with the IDC, while Natural shrublands, cropland, and pasture mosaics, rivers and water bodies, and clean pastures significantly contribute to higher IDC values (p < 0.050) (Table 3).

**Table 3:** Step-wise multiple regression model

Residuals:	Min	1Q	Median	3Q	Max
	-0.17760	-0.04293	-0.00521	0.02989	0.21350
Variables/Coefficients	Estimate	Std. Error	t value	Pr( >|t|)	Significance (< 0.050)
Intercept	0.43586	0.007971	54.683	< 2e^−16^	*
Sugarcane plantations	-0.26907	0.010776	-24.970	< 2e^−16^	*
Dense forest	-0.24005	0.014356	-16.721	< 2e^−16^	*
Urban areas	-0.40903	0.027881	-14.671	< 2e^−16^	*
Natural grasslands (Paramo)	-0.14953	0.016800	-8.901	< 2e^−16^	*
Natural shrublands	0.24755	0.031203	7.934	< 1.45e^−14^	*
Cropland and pasture mosaic	0.13680	0.031376	4.360	< 1.58e^−05^	*
Rivers and natural water bodies	0.90214	0.256902	3.512	0.000486	*
Clean pastures	0.06582	0.027501	2.394	0.017062	*
Weeded pastures	-0.07655	0.042348	-1.808	0.071252	
Cropland mosaic	0.25399	0.175123	1.450	0.147594	

Dependent variable: IDC. Predictive variables: 22 land covers. n: 502. Multiple R-squared: 0.7433. Adjusted R-squared: 0.7381. Residual standard error: 0.06372 on 491 degrees of freedom. F-statistic: 142.2 on 10 and 491 DF. p-value: < 2.2e^−16^

Significance codes: * < 0.050

These relationships reflect the distinct levels of ecological disturbances exerted by various agricultural practices of the region resulting in a land cover and land-use intensity spatial gradient (Fig. 2f). In this sense, the IDC revealed the existence of at least three types of landscape-metabolic configurations of the UCRV landscapes (Fig. 2f). The first type (*anthropic*) is defined by a very low IDC (0.05 < IDC < 0.3). This type depicts landscapes resulting from an industrial agriculture metabolism and is mainly concentrated along the river valley's flat area. The second type (*natural areas*) is defined by low-to-moderate IDC, low anthropogenic activity, low land cover heterogeneity, predominantly dense forests, and paramos. These areas are located mainly above the 3000 m a.s.l, where population density and activity are very low, usually associated with subsistence agriculture. A high IDC defines the third type of landscape-metabolic configuration (mosaics). It reflects heterogeneous landscapes with less intensive agricultural activities (intermediate disturbance levels). These landscapes are found at mid-elevations (1,200 m a.s.l. to 2,800 m a.s.l.) on the slopes of the Andean Mountain ranges. This area comprises agricultural, agroforestry, and agropastoral mosaics (Fig. 1), reflecting different agricultural practices, including traditional peasant, Afro, and Indigenous agroecological models (Duarte Torres et al. 2018). The findings build upon those of Marull et al. (2017) by extending the study beyond the administrative boundaries of a single municipality to encompass the Cauca River Valley region. This approach highlights the importance of adopting a green infrastructure approach within the metropolitan region of Cali and incorporating a broader spectrum of biocultural landscapes representative of the entire biogeographic region.

**Fig. 4:**
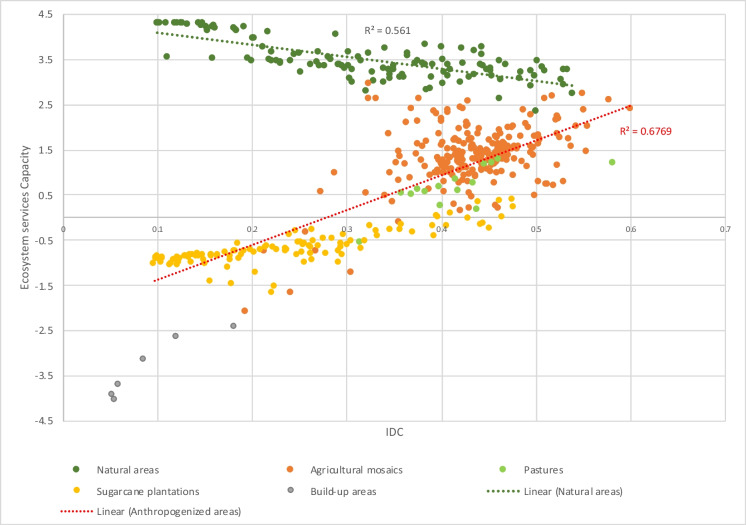
Relationship between the Intermediate Disturbance Complexity (IDC) model and the Ecosystem Services Capacity (ESC) in the metropolitan region of Cali. Note: The points represent each of the 502 landscape-scale analysis units. Anthropogenized areas: pastures, agricultural mosaics, sugarcane plantations, built-up areas. Natural areas: forests, grasslands, and shrubs. Colours indicate the predominant land cover in each unit of analysis (i.e., those occupying more than 50% of the total hexagon area): Natural areas (i.e., forests, grasslands, and shrublands), Agricultural mosaics, sugarcane plantations, and build-up areas (i.e., urban, suburban, and industrial)

The significance of integrating findings from this landscape-metabolism approach into the strategic planning of sustainable metropolises, particularly within socioeconomically intricate regions like the tropical Andes, is underscored by its contextualization within the land-sharing and land-sparing discourses (Fischer et al. 2014, 2008; Grass et al. 2019). The proposed classification of landscape metabolic configurations resulting from the IDC analysis, identifies, on one side, *anthropic* landscape-metabolic configuration is characteristic of a productive paradigm that has often been associated with land-sparing strategies to balance biodiversity conservation and agricultural production (mainly for food but see Anderson-Teixeira et al. (2012)). Secondly, the natural landscape-metabolic configurations concentrated in protected areas host critical ecosystems and perform essential functions and services for natural and societal communities. However, these areas are highly disconnected, possibly affecting the effectiveness of a land-sparing strategy (Cannon et al. 2019; Edwards et al. 2021). Finally, the areas with landscape-metabolic configurations of mosaics would reflect a land-sharing strategy for food production and biodiversity conservation (Perfecto et al. 2009; Perfecto and Vandermeer 2008). These areas increasingly offer suitable habitats, higher biodiversity, and multiple ecosystem services (Loreau et al. 2003; Marull et al. 2019; Margalef 1973; Tscharntke et al. 2005), and play a crucial role in ecological connectivity (Fig. 3). To illustrate this, we explore the relationship between the IDC and the capacity of metropolitan landscapes to supply ecosystem services (ESC) (Fig. 4). Landscape-metabolic configurations related to agro-industrial activity are associated with a lower capacity to supply ecosystem services for the metropolitan population (yellow dots in Fig. 4). On the contrary, the agricultural mosaic revealed a higher capacity to supply ecosystem services such as water supply, food production, and flood regulation among others (orange dots in Fig. 4). Since the IDC is based on the theoretical assumption that agricultural landscapes can retain more farm-associated biodiversity at intermediate levels of human net primary production appropriation (Loreau et al. 2003; Marull et al. 2015; Montero et al. 2021), the IDC predictive power decreases as non-anthropogenic land covers (i.e., natural forest, shrublands, pasturelands, and paramos) increases, as seen in Fig. 3. Accordingly, metropolitan green infrastructure must contain different interrelated and connected elements to provide a structure that provides distinct functions and services (Basnou et al. 2020; Hansen and Pauleit 2014). The ESC of landscapes is expressed along the land-use intensity gradient, with the lower capacity index where urban and sugarcane land covers are predominant (Fig. 2d). This is expected, as these land covers have a high demand for ecosystem services while offering none (i.e., urban) or very few (i.e., sugarcane monocultures) ecosystem services, for instance, the capacity to provide energy in the form of biomass. In contrast, the strip of intermediately disturbed well-connected and heterogeneous landscapes (higher IDC values) shows higher capacity and multifunctionality to supply ecosystem services.

We propose that the role of these biocultural agricultural mosaics is increasingly pertinent to the ongoing process of metropolitan growth in this region. While the nuances of the effectiveness of the land-sharing or land-sparing strategies go beyond the purpose and scope of this study and have already been widely revised in the literature (Fischer et al. 2017, 2014; Grau et al. 2013; Kremen 2015; Scariot 2013), their postulates can complement our analysis. The UCRV reflects the necessity of combined land-sharing and land-sparing strategies, the integrative assessment exposes that the effectiveness of these two strategies would be highly compromised by the poor ecological connectivity of the area. Our assessment reveals an important emerging category of metropolitan open spaces: the agrosilvopastoral mosaics. These biocultural landscapes hold a high capacity to supply ecosystem services and high ecological connectivity and help structure a well-connected, multifunctional green infrastructure (Table 4 and Table 5). Therefore, land use planning policies should consider the socioecological impacts of maintaining large agricultural areas with low levels of provision of ecosystem services (i.e., intensive sugarcane cultivation) at the cost of losing the ecological quality of the Cauca Valley by decreasing the provision of other essential ecosystem services and condemning the most populated area of the UCRV to ecological isolation and environmental degradation (Kremen and Miles 2012).

**Table 4:**
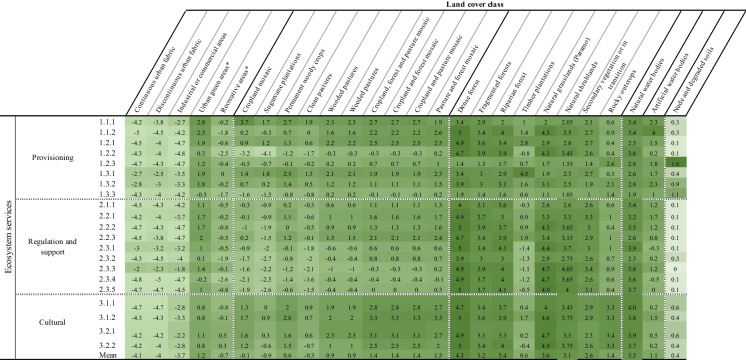
Ecosystem service capacity of the land covers of the metropolitan region of Cali

^*^These land cover classes were not mapped in Fig. 1 given their low representation in the study area

**Table 5:**
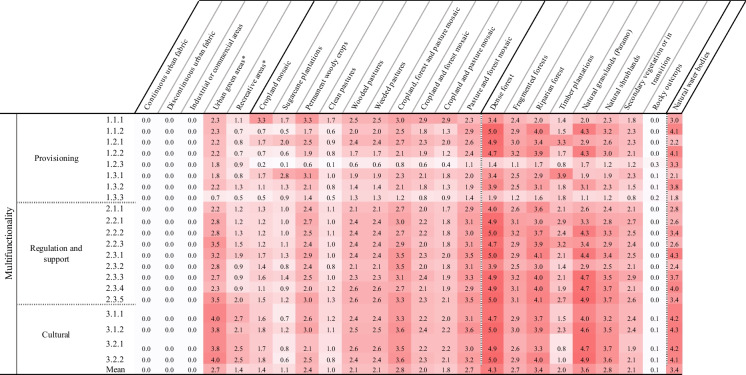
Ecosystem services multifunctionality of the land covers of the metropolitan region of Cali

^*^These classes were not mapped given their low representation in the study area

Our results show that the ecological rupture affects essential ecosystem flows between the paramos and high Andean Forest. This situation jeopardises the delivery of vital ecosystem services from these areas to the metropolis, including collecting, regulating, and providing water for human consumption and agriculture in the entire region (Table 4), especially, in a tropical Andes region where climatic phenomena, such as "El Niño," exacerbated by climate change trends, can bring extreme drought conditions, amplifying the risks of water shortages, crop failures, and floodings. These challenges highlight the urgent need for adaptive strategies and sustainable water resource management practices to mitigate the socio-economic and environmental impacts of these events and other manifestations of climate change in the region. Therefore, a systemic green infrastructure approach can play a pivotal role in both mitigation and adaptation efforts, offering nature-based solutions to enhance water retention, reduce flood risks, and promote ecosystem resilience in the face of changing climatic conditions.

Our results underscore the significance of preserving a diverse metropolitan green infrastructure and comprehending it not merely as the sum of its parts, but as a cohesive functional unit—a complex system wherein each component interacts through landscape-metabolic processes, giving rise to emergent properties that support various functions and services crucial for the metropolitan region. These functions include connectivity, complementarity, and the provision of ecosystem services. Furthermore, the proposed systemic perspective interprets Cali's process of metropolization as the interaction between the built environment and green infrastructure, from which humans derive benefits. This perspective integrates aspects of green infrastructure as a subsystem into metropolitan urban analyses, recognizing its capacity to provide benefits to society (Tabares-Mosquera et al. 2020).

These results confirm the importance of polyculture landscapes and different types of forests (dense and riparian), which not only provide resources to metropolitan areas but also regulate ecosystem functions like supporting life cycles, protecting habitats and gene pools, pest control, and soil health (Bennett and Radford 2008; Grass et al. 2019; Kennedy et al. 2013; Tscharntke et al. 2012). These findings are pertinent to land scarcity discussions and can inform analyses of conflicts related to biofuel, food, and biodiversity production (Fischer et al. 2014; Lambin and Meyfroidt 2011). Thus, it's crucial to explore various agricultural practices (conventional, organic, agroecological) at both farm and landscape scales, as they can significantly influence agroecosystem functioning and contribute to landscape sustainability (Guzmán et al. 2011) and ecosystem services (Padró et al. 2020a).

### Importance of the metropolitan green infrastructure for land use planning

In this study, metropolitan green infrastructure is defined as the collection of natural and semi-natural land covers whose condition, extent, and connectivity allow them to provide ecosystem services (Tabares-Mosquera et al. 2020). The results derived from the IDC model reveal a gradient of landscape-metabolic configurations within the metropolitan green infrastructure. These configurations, shaped by the intricate interactions among different green infrastructure elements, align with the IDC principles, where higher IDC values correlate with increased capacity and multifunctionality in providing ecosystem services (Marull et al. 2015). This gradient-based approach emphasizes the varied roles that landscape components play in supplying ecosystem services (Jones et al. 2013), shedding light on the complex relationship between these elements within the metropolitan area. For instance, the COVID-19 pandemic made evident the food sovereignty challenges of a metropolitan region (Rankin et al. 2021) isolated in landscape-metabolic configurations resulting from sugarcane industrialized monocultures, and it became evident that this homogeneous landscape-metabolic territorial set-up reduces the ecosystem service supply, and ultimately, the resilience of the entire metropolitan system.

Lastly, our research provides key insights into sustainable landscape patterns (Peng et al. 2021). The results support the idea that complex and heterogeneous landscape configurations can enhance ecosystem services while promoting landscape connectivity and multifunctionality, underscoring the intricate interactions among green infrastructure elements, landscape sustainability and resilience in metropolitan regions.

To offer valuable guidance for policy-makers and land planners aiming to develop strategies that enhance long-term sustainability at the landscape level, with a focus on promoting landscapes that are both ecologically robust and socially beneficial, we propose five reflections to guide land-use planning discussions in the Upper Cauca River Valley (UCRV) and other metropolitan regions in the tropical Andes, particularly those facing challenges from land-use change and intensification.

#### Adopting a landscape approach

Adopting the landscape scale for urban and territorial planning in metropolitan systems is increasingly relevant for land planning. In the first place, many of the ecosystem functions and services of the metropolises occur at this scale, which is key to overcoming the methodological difficulties of their evaluation, monitoring, and incorporation into public policy. The landscape scale allows closing metabolic cycles (water, energy, food, waste,) necessary to move towards a more circular economic model, which implies fostering interactions between built and open spaces, relationships of great relevance to society (Bennett and Radford 2008; Tello et al. 2017). Finally, considering the landscape as one of the essential background elements for the sustainability of the metropolitan system allows, under the right conditions, the reproduction of biophysical flows essential for its sustainability, such as those related to the agri-food system (Cattaneo et al. 2018; Marull et al. 2016a).

#### Re-establish the functional structure of the landscape

Restoring landscape complexity (i.e., heterogeneous, and well-connected land covers) would imply a substantial and long-term productive transformation of the valley. However, interstitial spaces in the flat plain can be a starting point for reconfiguring the territory and promoting an agricultural mosaic with a high capacity to provide ecosystem services. Improving the ecological structure of pastureland covers (i.e., promoting wooded pastures) would be crucial because of their affinity with agroforestry mosaics and heterogeneous crops, serving as stepping stones for ecological processes.

#### Enhancing ecological connectivity and protecting high mountain nature

Altitudinal gradients are associated with highly endemic biodiversity (Larsen et al. 2009). Therefore, a well-connected network of natural areas should facilitate the altitudinal migration of species threatened by global warming, anthropogenic habitat loss, and degradation (Balthazar et al. 2015; Cresso et al. 2020; Lambin et al. 2003). For instance, despite occupying less than 8% of the study area, the land covers associated with the Páramos ecosystem (i.e., grasslands and natural grasslands) have a high capacity to supply essential ecosystem services. A region with good connectivity at the macro-basin scale will likely counteract the impact suffered by the hydrological system of the UCRV.

#### Configure mosaic landscapes following agroecological management

The results show the great weight that the agrosilvopastoral mosaic area exerts on the provision of ecosystem services and the ecological connectivity of the metropolis. However, these values may vary depending on the type of agriculture practised (e.g., conventional, organic, agroecological) (Font et al. 2020; Marull et al., 2020; Padró et al. 2020b). Given this condition, the international consensus points to a necessary global agroecological transition, for which Latin America plays a fundamental role (Altieri and Nicholls 2012; Altieri and Toledo 2011; Jeanneret et al. 2021; Perfecto and Vandermeer 2008). The priorities should focus on improving the metropolitan areas' capacity to close metabolic cycles (Billen et al. 2021; Cattaneo et al. 2018) and providing multiple ecosystem services. This can be achieved in combination with promoting highly multifunctional land uses, where crop and livestock systems are integrated. The potential of agricultural mosaics for sustainable land use planning and management in metropolitan regions is critical (Tscharntke et al. 2021).

#### Define and implement a metropolitan green infrastructure

Based on the five previous elements, both the needs and the opportunities to adopt a conceptual and methodological green infrastructure framework to face the sustainability challenges of the UCRV are evident. The results are in line with cutting-edge studies on urban and territorial planning of metropolitan areas, which increasingly reinforce the need to include agricultural open spaces as fundamental elements for the sustainability of the metropolitan system given its multifunctional character (Basnou et al. 2020; Slätmo et al. 2019; Yacamán-Ochoa et al. 2020). This implies defining a network of interconnected open spaces, including peri-urban and rural spaces and natural spaces, capable of providing diverse ecological services, goods, and functions for society. The agrarian elements of this green infrastructure are fundamental, and, therefore, it is recommended to include them in the landscape and urban planning of metropolitan regions.

## Conclusions

This article presents a territorial-metabolic approach for studying landscape sustainability and offers a framework that explores the interconnectedness of landscape configuration, ecosystem services, and, ultimately, human well-being. The approach integrates landscape-metabolism to assess how urban growth and agricultural intensification impact the socioecological functionality of a tropical Andean metropolitan region. Our results elaborate on Marull et al. 2017, by providing a classification of landscape metabolic configurations, and relating them with the provision of ecosystem services (i.e., water supply, food production, flood regulation among others), increasingly pertinent to the ongoing process of metropolitan growth in this region and the occurrence of regular yet increasingly severe climatic phenomena. Different landscape-metabolic configurations along a land-use gradient are linked to ecosystem service provisioning. The findings reveal that the current land use planning model, driven by agricultural intensification and industrialization, has degraded the metropolitan socioecological quality, undermining sustainable urban and rural progress. The proposed landscape-metabolism model aims to guide decisions in land use planning and territorial management to enhance sustainability and the multifunctionality of biocultural landscapes for ecosystem service provision.

The Intermediate Disturbance Complexity (IDC) model, originally validated in Mediterranean ecosystems, was applied in a tropical Andean region. Evaluating ESC in such intricate landscapes presents challenges, especially in economically developing nations constrained by resources. However, the IDC model offers a methodologically straightforward solution, utilizing a land cover map and data on metropolitan green infrastructure metabolism (e.g. agricultural production and management) to compute an indicator closely linked to ESC and multifunctionality. The IDC model distinguishes itself for its efficiency and efficacy in capturing not only landscape dynamics but also furnishing insights into landscape configuration and social metabolism, allowing researchers and land planners to understand the importance of agroecological landscapes for socioecological sustainability (Tello & González de Molina 2023).

Future research should prioritize assessing synergies and trade-offs in territorial planning to address multiple socio-ecological challenges, such as enhancing ecological connectivity in the valley's hydrological network and mitigating conflicts over water for human consumption and agriculture. Given the prevalence of inequality and poverty in tropical Andes metropolitan regions and Latin America, future socio-ecological studies should also incorporate environmental justice considerations into their assessments and objectives.

## Data Availability

The data used in this study is available upon request by contacting the authors directly.

## Footnotes

1 The population figures provided for the Upper Cauca River Valley in this study correspond to the aggregated municipal population reported by the 2018 National Census for the 25 municipalities considered part of the UCRV region in this study. Municipalities: Buenos Aires, Caldono, Cali, Caloto, Candelaria, Corinto, El Cerrito, Florida, Ginebra, Guacari, Guachene, Jambaló, Jamundí, Miranda, Morales, Padilla, Palmira, Pradera, Puerto Tejada, Santander de Quilichao, Suarez, Toribío, Vijes, Villa Rica, Yumbo,
